# Electroacupuncture Promotes Recovery of Motor Function and Reduces Dopaminergic Neuron Degeneration in Rodent Models of Parkinson’s Disease

**DOI:** 10.3390/ijms18091846

**Published:** 2017-08-24

**Authors:** Jaung-Geng Lin, Chao-Jung Chen, Han-Bin Yang, Yi-Hung Chen, Shih-Ya Hung

**Affiliations:** 1 School of Chinese Medicine, China Medical University, Taichung 40402, Taiwan; jglin@mail.cmu.edu.tw; 2 Graduate Institute of Integrated Medicine, College of Chinese Medicine, China Medical University, Taichung 40402, Taiwan; cjchen@mail.cmu.edu.tw; 3 Graduate Institute of Acupuncture Science, China Medical University, Taichung 40402, Taiwan; alex23206567@gmail.com; 4 Department of Photonics and Communication Engineering, Asia University, Taichung 41354, Taiwan; 5 Research Center for Chinese Medicine and Acupuncture, China Medical University, Taichung 40402, Taiwan; 6 Division of Colorectal Surgery, China Medical University Hospital, Taichung 40447, Taiwan

**Keywords:** dopamine, electroacupuncture, motor function, neuroprotection, Parkinson’s disease

## Abstract

Parkinson’s disease (PD) is a common neurodegenerative disease. The pathological hallmark of PD is a progressive loss of dopaminergic neurons in the substantia nigra (SN) pars compacta in the brain, ultimately resulting in severe striatal dopamine deficiency and the development of primary motor symptoms (e.g., resting tremor, bradykinesia) in PD. Acupuncture has long been used in traditional Chinese medicine to treat PD for the control of tremor and pain. Accumulating evidence has shown that using electroacupuncture (EA) as a complementary therapy ameliorates motor symptoms of PD. However, the most appropriate timing for EA intervention and its effect on dopamine neuronal protection remain unclear. Thus, this study used the 1-methyl-4-phenyl-1,2,3,6-tetrahydropyridine (MPTP)-lesioned mouse model (systemic-lesioned by intraperitoneal injection) and the 1-methyl-4-phenylpyridinium (MPP^+^)-lesioned rat model (unilateral-lesioned by intra-SN infusion) of PD, to explore the therapeutic effects and mechanisms of EA at the GB34 (Yanglingquan) and LR3 (Taichong) acupoints. We found that EA increased the latency to fall from the accelerating rotarod and improved striatal dopamine levels in the MPTP studies. In the MPP^+^ studies, EA inhibited apomorphine induced rotational behavior and locomotor activity, and demonstrated neuroprotective effects via the activation of survival pathways of Akt and brain-derived neurotrophic factor (BDNF) in the SN region. In conclusion, we observed that EA treatment reduces motor symptoms of PD and dopaminergic neurodegeneration in rodent models, whether EA is given as a pretreatment or after the initiation of disease symptoms. The results indicate that EA treatment may be an effective therapy for patients with PD.

## 1. Introduction

Parkinson’s disease (PD) is the second most common neurodegenerative disorder after Alzheimer’s disease, affecting an estimated 7 to 10 million patients worldwide [1]. A recent systematic review of studies of PD incidence reported overall incidence rates of PD in male and females aged ≥40 years of 61.21 (95% confidence interval 43.57–85.99) and 37.55 (95% confidence interval 26.20–53.83) per 100,000 person-years, respectively [2]. The incidence increases with age in both sexes [2]. PD is characterized by substantial (50–70%) loss of dopaminergic neurons in the substantia nigra (SN) pars compacta that project to the striatum [3]. A large number of motor and non-motor features including rest tremor, bradykinesia, rigidity, and loss of postural reflexes are considered to be the cardinal signs of PD [4]. Genetics and environmental risk factors have been implicated in the etiology of PD, but the primary cause remains unknown [5]. PD is one of the most disabling chronic neurologic diseases and leads to a significant loss of quality of life [6]. The main types of medications available to treat symptoms of PD include: levodopa, dopamine agonists, monoamine oxidase type B (MAO-B) inhibitors, and catechol-*O*-methyltransferase (COMT) inhibitors, amantadine, and anticholinergics [7,8]. Initially, drug therapy may significantly improve PD symptoms, but the benefits frequently wear off over time or become less consistent [8]. Deep brain stimulation (DBS) surgery has been shown to improve motor symptoms and quality of life in patients with advanced stages of PD [9]. However, the procedure is used only for patients whose symptoms cannot be adequately controlled with medications and most patients still need to take medication after undergoing DBS.

Acupuncture represents a non-invasive complementary therapy and has long been used in traditional Chinese medicine to treat PD, described in ancient Chinese literature as a “trembling” and “convulsive disease”; in particular, acupuncture is used to alleviate the symptoms of tremor and pain [10]. In China, acupuncture is a treatment of choice for many clinicians and PD patients [11]. Benefits of acupuncture treatment include significant increases in the total Unified Parkinson Disease Rating Scale score of PD patients [12]. Moreover, following acupuncture treatment, 70–80% of patients reported improvements in subjective symptoms, motor scores, and significant amelioration of their sleep and mood [13,14,15,16,17].

Electroacupuncture (EA) has become increasingly popular since the 1970s and is often the preferred from of acupuncture among researchers, as it is easier to standardize the type and amount of stimulation administered to each subject in comparison to classical manual acupuncture. The results of a recent pilot study demonstrate improvements in rigidity and balance in 15 PD patients administered EA treatment [18]. EA at the Baihui (GV20) and Dazhui (GV14) acupoints attenuates rotational behavior in rats and increases survival rates of dopaminergic neurons [19,20]. However, it remains unclear whether these effects are specific to acupoint electrostimulation or due to non-specific electrostimulation. In mice, EA stimulation improved behavioral deficits, protected dopaminergic neurons, and augmented striatal dopamine content [21]. In this 1-methyl-4-phenyl-1,2,3,6-tetrahydropyridine (MPTP) mouse model of PD, MPTP is systemically injected intraperitoneally (i.p.) to produce a loss of dopamine nerve terminals, which are not specific to the nigrostriatal pathway. Thus, mechanisms underlying EA-associated improvements in PD symptoms are yet to be elucidated.

1-Methyl-4-phenylpyridinium (MPP^+^), the active metabolite of the neurotoxin 1-methyl-4-phenyl-1,2,3,6-tetrahydropyridine (MPTP), inhibits NAD(H)-linked mitochondrial oxidation at the level of Complex I of the electron transport system [22,23]. In the MPTP mouse model of PD, MPTP is injected i.p. to kill dopaminergic neurons and produce dopamine deficiency in nerve terminals. The effect is not specific to the dopamine nerve terminal degeneration of the substantia nigra, but to all dopamine nerve terminals throughout the whole brain. In the nigrostriatal pathway, nerve terminals in the striatum project from the nigral dopaminergic neurons. In the MPP^+^ rat model of PD, MPP^+^ is unilaterally injected into the SN by direct infusion to produce unilateral depletion of dopamine in the striatum. One of the most attractive features of the unilateral MPP^+^ model is that each animal can serve as its own control (the opposite side serves as an intra-animal control) as there is a lesioned and an un-lesioned hemisphere [24]. After unilateral depletion of dopamine, tyrosine hydroxylase positive dopaminergic neurons can be analyzed. The unilateral lesion model is particularly useful for certain motor tests in behavioral analyses such as apomorphine-induced rotation, reaching task, tactile placing, etc. [25].

The ability to set stimulation frequency and intensity with EA enables objective and quantifiable measurements, which are superior to manual acupuncture during treatment [26]. Most reports have used i.p. injections of MPTP in mice to evaluate the therapeutic effects of manual acupuncture for 15 s at the GB34 and LR3 acupoints [21,27,28]. In one study, acupuncture at GB34 and LR3 decreased in tyrosine hydroxylase immunoreactivity and generated neuroprotective effects in the SN and striatum on days 1, 3, and 7 post-MPTP injection in mice [21]. Only a few studies have used unilateral lesion by intrastriatal injection or medial forebrain bundle as the PD rat model. One study showed that manual acupuncture for 15 s at GB34 and LR3 inhibits neurotoxicity and rotational behavior in the PD rat model 2 weeks after intrastriatal injection of 6-hydroxydopamine [29]. No reports are available as to the therapeutic effects of EA in parkinsonian animals after unilateral lesion induced by intra-SN injection. It remains unclear as to whether electrical stimulation by EA exerts neuroprotective effects against MPP^+^-induced dopaminergic neuronal death in vivo. This study aimed to determine whether electrical stimulation of dopaminergic neurons assists with functional recovery in the MPP^+^-treated model of PD.

## 2. Results

### 2.1. Electroacupuncture (EA) Improved Motor Symptoms of Parkinson’s Disease (PD) and Ameliorated Dopaminergic Neurodegeneration in 1-Methyl-4-phenyl-1,2,3,6-tetrahydropyridine (MPTP) Mice Models

**Figure 1:**
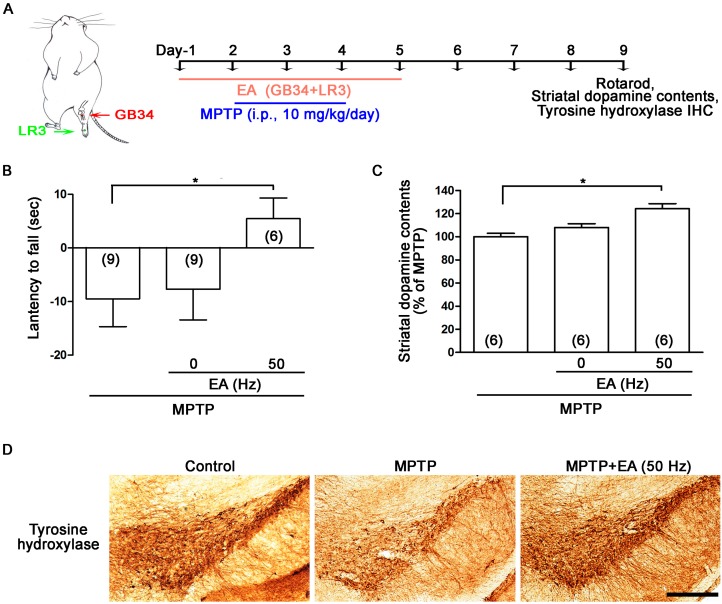
Electroacupuncture (EA) reduced motor deficiency and dopaminergic neurodegeneration in 1-methyl-4-phenyl-1,2,3,6-tetrahydropyridine (MPTP) mice. Systemic MPTP injections (intraperitoneal; i.p., 10 mg/kg/day for three days) induced dopaminergic neuronal loss in the substantial nigra (SN) and striatal nerve terminal degeneration in both sides of the nigrostriatal dopaminergic pathway. (**A**) The experimental procedure of EA at the GB34 (Yanglingquan) and LR3 (Taichong) acupoints in MPTP mice; (**B**) Rotarod results on the last day (day 9) showed that MPTP treatment reduced the latency to fall as compared to baseline (before treatment) on day 1. EA stimulation at 50 Hz but not 0 Hz significantly increased the latency to fall in the rotarod test on day 9; (**C**) Dopamine content from both sides of the mouse brain showed that EA 50 Hz but not 0 Hz stimulation increased the striatal dopamine content as compared to MPTP-alone treated animals; (**D**) Immunohistochemistry (IHC) analysis of tyrosine hydroxylase levels in the SN also indicated that 50 Hz EA stimulation exerted neuroprotection in dopaminergic neuronal bodies. Scale bar = 300 μm. *, *p* < 0.05 compared with MPP^+^-treated group.

MPTP is the most commonly used parkinsonian neurotoxin. Systemic MPTP injections (i.p., 10 mg/kg/day for three days) induced substantial nigra (SN) dopaminergic neuronal loss and nerve terminal degeneration in the striatum on both sides of the nigrostriatal dopaminergic pathway. The experimental EA procedure at the GB34 and LR3 acupoints in MPTP mice is illustrated in Figure 1A. On the last day (day 9), rotarod results showed that MPTP treatment reduced the latency to fall compared to baseline values (before treatment) on day 1 (Figure 1B). EA stimulation at 50 Hz but not 0 Hz significantly increased the latency to fall in the rotarod test on day 9 (Figure 1B). Dopamine contents from both sides of the mouse brain showed that EA 50 Hz but not 0 Hz stimulation increased the striatal dopamine content by LC-MS/MS analysis as compared to MPTP (Figure 1C). Immunohistochemistry (IHC) data of tyrosine hydroxylase also indicated that 50 Hz EA stimulation exerted neuroprotection in the dopaminergic neuronal body in the SN (Figure 1D). These data indicate that pre-treatment with EA at 50 Hz but not 0 Hz exerts motor improvement and neuroprotection in the MPTP mouse model.

### 2.2. EA Improved Motor Asymmetry in MPP^+^-Lesioned Rats

**Figure 2:**
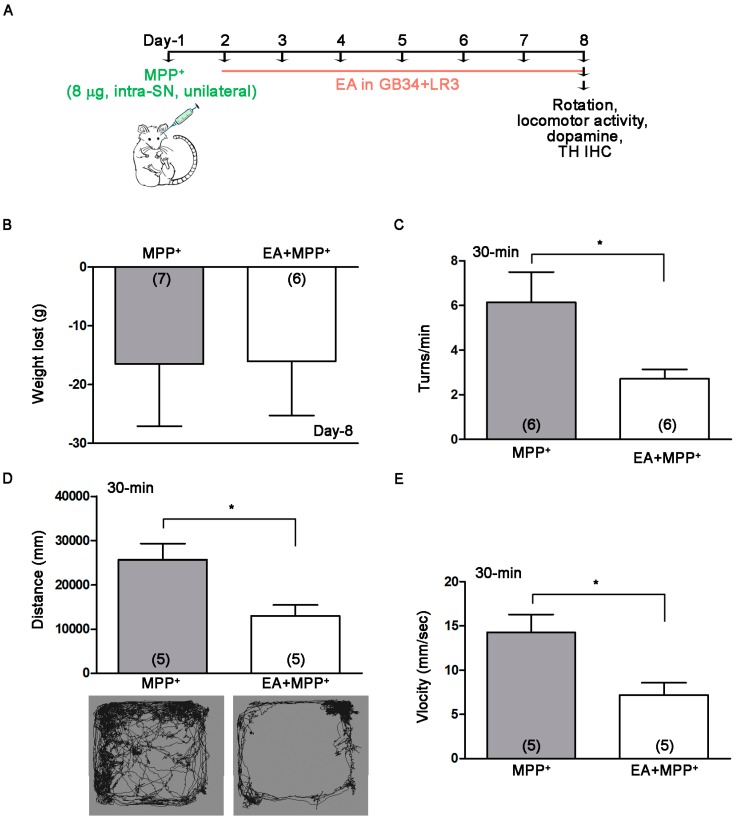
EA reduced motor deficiency in rats unilaterally lesioned with MPP^+^. Intracerebral injection of MPP^+^ (8 μg) into one side of substantia nigra (SN) induces dopaminergic neuronal death in the SN and depletes striatal dopamine. In this study, EA was applied to rats after MPP^+^ administration. (**A**) shows the experimental protocol of the rat study. Eight days after MPP^+^ administration, EA did not increase MPP^+^-induced body weight loss (**B**), but significantly reduced apomorphine-induced turning behavior (**C**) and locomotor activity, as assessed by both distance and velocity (**D**). The data indicate that 50 Hz EA stimulation at GB34 and LR3 acupoints after MPP^+^ administration reduced motor deficiency in rats unilaterally lesioned with MPP^+^. *, *p* < 0.05 compared with MPP^+^-treated group.

1-Methyl-4-phenylpyridinium (MPP^+^) is the toxic metabolite of MPTP. Intra-cerebral infusion of MPP+ into one side of the SN induces dopaminergic neuronal death leading to dopamine depletion in the striatum. In this study, rats were treated with EA after undergoing MPP^+^ (8 µg). Figure 2A shows the experimental protocol of the rat study. EA 0 Hz stimulation at the GB34 and LR3 acupoints had no effect upon motor dysfunction and dopaminergic neurodegeneration in MPTP mice (Figure 1). We subsequently applied EA 50 Hz stimulation in rats at the GB34 and LR3 acupoints on the day after MPP^+^ injection. Apomorphine is a dopamine receptor agonist that induces locomotor activity in 6-hydroxydopamine-treated rats [30]. In our previous study, apomorphine (5 mg/kg, i.p.) did not induce rotation behavior in untreated controls [24]. In this study, at eight days after MPP^+^ administration, EA did not increase MPP^+^-induced body weight loss but did significantly reduce apomorphine-induced turning behavior and locomotor activity, as assessed by both distance and velocity (Figure 2B−E). These results indicate that EA 50 Hz stimulation at the GB34 and LR3 acupoints reduces motor asymmetry in MPP^+^-lesioned rats.

### 2.3. EA Provides Dopaminergic Neuroprotection in MPP^+^-Lesioned Rats

**Figure 3:**
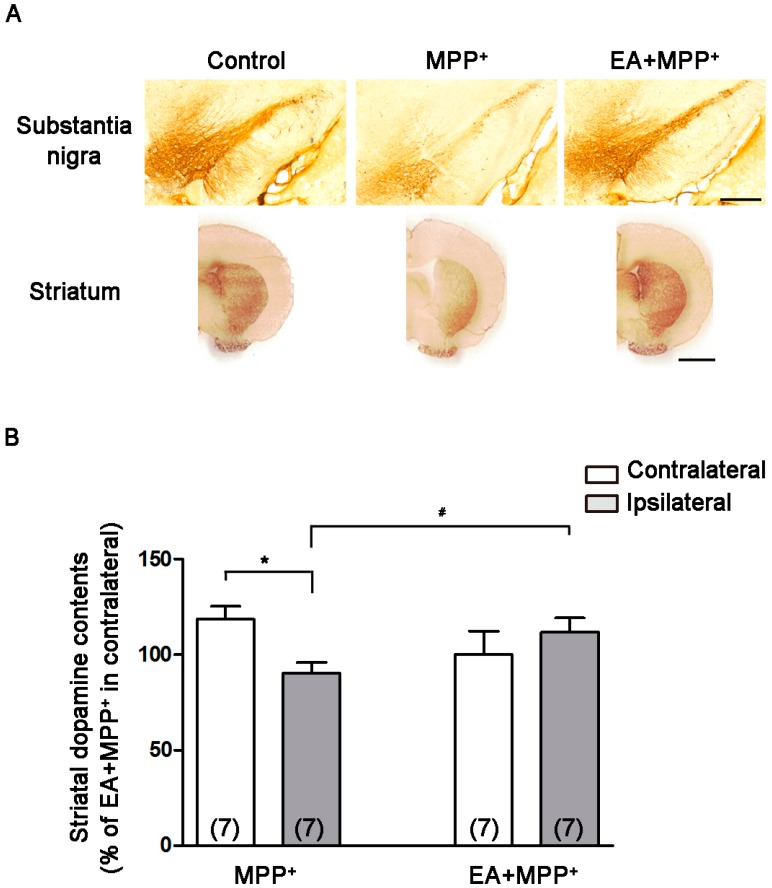
EA produces dopaminergic neuroprotection in rats unilaterally lesioned with MPP^+^. Immunostaining for tyrosine hydroxylase in the rat substantia nigra (SN) and striatum was performed eight days after MPP^+^ lesions. (**A**) shows EA 50 Hz stimulation repressed tyrosine hydroxylase (+) neuronal loss in the SN and induced dopaminergic terminal degeneration in the striatum. Scale bars = 200 μm (top) and 2 mm (bottom); (**B**) Unilateral MPP^+^ lesioning significantly reduced striatal dopamine content in the ipsilateral side of the injection as compared to the contralateral side (Figure 3B). *, *p* < 0.05 compared with contralateral side of MPP^+^; #, *p* < 0.05 compared with MPP^+^-treated group.

Immunostaining for tyrosine hydroxylase in rat SN and striatum was performed eight days after MPP^+^ intra-SN injection. Figure 3A shows that EA 50 Hz stimulation reduced the loss of tyrosine hydroxylase-positive neurons in the SN and degeneration in striatal terminal. MPP^+^-lesioned rats had significantly reduced striatal dopamine content in the ipsilateral side compared with the contralateral striatum (Figure 3B). These data are consistent with our previous study [24]. Following MPP^+^ injection, EA stimulation reduced dopamine depletion in the ipsilateral side (Figure 3B). These data suggest that EA stimulation after MPP^+^-lesioning provides dopaminergic neuroprotection in rats.

### 2.4. EA Reduced MPP^+^-Induced Dopaminergic Neuronal Apoptosis via Increasing Brain-derived Neurotrophic Factor (BDNF) Expression and Further Akt Phosphorylation in Rat Substantia Nigra (SN)

**Figure 4:**
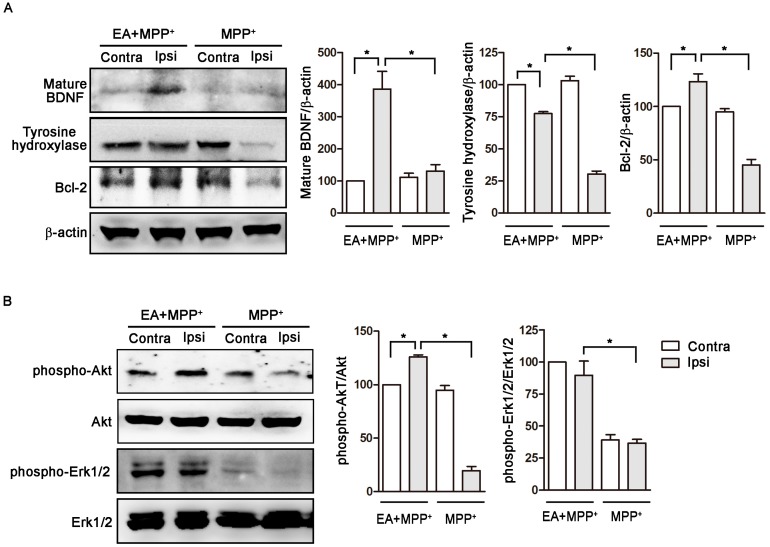
EA reduced MPP^+^-induced dopaminergic neuronal apoptosis by increasing BDNF (brain-derived neurotrophic factor) expression and further Akt phosphorylation in the rat substantia nigra. (**A**) Eight days after MPP^+^ administration, our Western blot results show that MPP^+^ treatment reduced tyrosine hydroxylase and Bcl-2 expression in the ipsilateral side of the rat substantia nigra (SN), but not in the contralateral side. EA stimulation (50 Hz) enhanced mature BDNF, tyrosine hydroxylase, and Bcl-2 expression in the MPP^+^-treated ipsilateral side; (**B**) EA enhanced the phosphorylation of Akt (phospho-Akt) but had no effect on Erk1/2 phosphorylation (phospho-Erk1/2) in the MPP^+^-treated ipsilateral side (Figure 4B). The data suggest that the way in which EA exerts dopaminergic neuroprotection in the unilateral MPP^+^-lesion model is by activating Akt phosphorylation and enhancing BDNF and Bcl-2 expression in the rat SN. * *p* < 0.05 compared with contralateral side of EA+MPP^+^ or ipsilateral side of EA+MPP^+^ group.

Brain-derived neurotrophic factor (BDNF) enhances the survival and morphological differentiation of dopaminergic neurons, whereas anti-BDNF neutralizing antibody enhances dopaminergic neuronal death [31]. The serine/threonine kinase Akt is required for neuronal survival and can be activated by BDNF. MPTP treatment reduces phospho-Akt expression in the mouse midbrain [32]. Bcl-2 overexpression reduces MPP^+^-induced cell death in MN9D cells in vitro [33]. Eight days after MPP+ administration, our Western blot results show that MPP^+^ treatment reduced tyrosine hydroxylase levels and Bcl-2 expression in the ipsilateral side (Ipsi) of rat SN but not in the contralateral side (Contra) (Figure 4A). These findings indicate that MPP^+^-induced dopaminergic neuronal death (tyrosine hydroxylase ↓) in rat SN involves neuronal apoptosis (Bcl-2 ↓), which is consistent with our immunohistochemical analyses of tyrosine hydroxylase, as shown in the upper part of Figure 3A. EA stimulation (50 Hz) enhanced the expression of mature BDNF, Bcl-2, and tyrosine hydroxylase levels in the ipsilateral side (Figure 4A). Furthermore, EA enhanced the phosphorylation of Akt but had no effect on Erk1/2 phosphorylation in the ipsilateral side (Figure 4B). These data suggest that EA provides dopaminergic neuroprotection in the unilateral MPP^+^-lesion model by enhancing BDNF and Bcl-2 expressions with activation of the Akt survival pathway in rat SN.

## 3. Discussion

In this report, we used two different PD animal models to evaluate the effects of EA on motor improvement (symptoms) and dopaminergic neuron protection. First, we used the MPTP (systemic-injected) mouse model as an initial screening tool to test EA in treating motor deficiency in PD. We found that pretreatment with EA 50 Hz but not 0 Hz at the GB34 and LR3 acupoints improves motor symptoms, enhances survival of dopaminergic neuron in the SN, and prevents the functional loss of striatal dopaminergic nerve terminal (in the striatum). In the MPP^+^ rat model of rats (caused by unilateral intra-SN injection), post-treatment with EA 50 Hz at the GB34 and LR3 acupoints reduced apomorphine-induced locomotor activity and turning behavior, and protected against dopaminergic neuronal degeneration and apoptosis by enhancing BDNF and Bcl-2 expressions and regulating the survival of neuronal cells through the activation of the Akt pathway. It is important to note that the increases in the expression of BDNF and phosphor-Akt in the SN were only correlated with EA treatment. Neurotoxin-based models produced by direct intrastriatal injection of 6-hydroxydopamine and systemic injections of MPTP are the most widely used PD models [25]. The unilateral lesion model is more complicated to perform than bilateral parkinsonism. Our study is the first to report on the use of the unilateral intra-SN MPP^+^-lesioned model in rats to evaluate the effects of EA in the treatment of PD.

Oxidative injury and mitochondrial dysfunction are major causes of neuronal death in PD. Bcl-2 is widely expressed in the developing brain and acts as an important regulator of cell death (from oxidative stress, lipid peroxidation, and compromised mitochondria respiration, etc.) in developing sympathetic neurons after neuronal growth factor deprivation [34,35]. In mice overexpressing Bcl-2, MPTP toxicity is attenuated via the enhancement of antioxidant activity and the suppression of apoptosis [34]. Our results show that Bcl-2 expression was reduced by MPP^+^ treatment and promoted by EA.

It remains unclear as to how signaling mechanisms mediate the effects of acupuncture in midbrain dopamine neurons. However, much evidence suggests that the key event of apoptosis in neurodegenerative disease is associated with levels of p53, and recent research has reported that the gene network associated with p53 signaling mediates the protective effects of acupuncture treatment in the mouse PD model [36]. Following MPTP injections, the application of acupuncture to the GB34 acupoint induced neuroprotective effects in the mouse striatum and SN, resulting in significant recovery to normal levels in the context of behavior and molecular signatures [36]. Notably, specific knockout of the p53 gene in the midbrain dopamine neurons abrogated the protective effects associated with acupuncture [36]. Thus, downregulating neuronal p53 activity may be a worthwhile therapeutic approach in PD. Besides effecting changes in signaling pathways, accumulating evidence suggests that the neuroprotective effects of acupuncture also involve endogenous biological mediators, such as the neurotrophin (NT) family of proteins, specifically, BDNF [37]. In a rat model of PD with unilateral 6-hydroxydopamine (6-OHDA) lesions in the medial forebrain bundle, four weeks of EA treatment at 100 Hz reversed the 6-OHDA-induced abnormal expression of BDNF on the lesioned side in the ventral midbrain and hippocampus [38]. BDNF may therefore serve as a potential therapeutic target in PD.

The Zusanli (ST36) and Sanyinjiao (SP6) acupoints are often used in the treatment of patients with PD, according to traditional Chinese medicine (TCM) principles [39]. Scientific investigations have shown that stimulation at these acupoints can enhance neuroprotection and improve mobility in PD [40]. In an investigation into the underlying mechanisms of this strategy, researchers found that 100 Hz EA stimulation at ST36 and SP6 in MPTP-lesioned mice elevated the Bcl-2/Bax ratio and produced anti-apoptotic effects [39]. A case study from Japan describes the successful treatment of an 81-year-old woman with PD who received manual acupuncture treatment five days per week based on TCM principles for the relief of non-motor symptoms (hot flashes and paroxysmal sweating, depression and anxiety), using the LR3, LI4, KI5, KI7, SP6, GB34, BL18, BL15, and GB20 acupuncture points [41]. A study that used functional magnetic resonance imaging (fMRI) to explore the effects of acupuncture stimulation at acupoint GB34 in patients with PD revealed the activation of the putamen and primary motor cortex [42]. This was accompanied by significant improvement of motor function, in finger-tapping tasks of the affected hand following acupuncture. A study conducted in China has reported good efficacy with the so-called “seven acupoints of cranial base” (SACB; consisting of DU15, bilateral BL10, GB20, and GB12) technique, when used as an adjunctive treatment with pharmacological therapy in patients with PD [43]. A review of data from 14 clinical studies involving a total of 519 patients with PD indicated potential benefits with acupuncture, especially for the treatment of non-motor symptoms [44]. However, most of the included studies had methodological flaws that limited the reliability of their results. Notably, there was a wide discrepancy amongst the studies as to which acupoints were used, making it difficult to interpret the results and compare the studies. In all 14 studies, approximately 40 body acupoints were used, but only LR3, GB34, ST36, and K3 were repeatedly chosen in a few studies; furthermore, the majority of acupoints were used only once or twice in treatment.

Currently, no validated acupuncture treatment protocol exists for PD, and the literature to date fails to provide any consensus regarding acupoint selection. Future acupuncture research in PD needs to identify more acupoints that are effective in PD treatment and agree on the selection of acupoints. Moreover, as none of the 14 studies included in the review provided long-term outcome data following treatment completion [44], evidence is needed on the sustained therapeutic effect of acupuncture. Future acupuncture research should follow accepted standards of methodology, such as CONSORT and STRICTA checklists, include sufficiently large sample sizes in clinical studies, and ensure reproducibility [44].

## 4. Materials and Methods

### 4.1. Animals

Male C57BL6 mice (4–8 weeks) and Sprague-Dawley (SD) rats (350 g; BioLasco Taiwan Co., Ltd, Taipei, Taiwan) were used. Animals were housed in our animal facility under a 12 h light/dark cycle with food and water available ad libitum for at least four days prior to the experiments. This study was carried out with the approval of the Institutional Animal Care and Use Committee (IACUC) of China Medical University on 30 December 2014 (104-194-N).

### 4.2. MPTP Intraperitoneal (i.p.) Injections in Mice and MPP^+^ Intra-Substantia Nigra (Intra-SN) Injection in Rats

Mice received i.p. injections of 10 mg/kg MPTP (1-Methyl-4-phenyl-1,2,3,6-tetrahydropyridine hydrochloride, Sigma-Aldrich (St. Louis, MO, USA), one injection daily for three days) 24 h after the first EA stimulation (Figure 1A). Rats received a unilateral injection of MPP^+^ (1-Methyl-4-phenylpyridinium iodide, 8 µg; Sigma-Aldrich) into the SN 24 h before the first EA stimulation (Figure 2A). The procedures for intra-SN injections and the setting of establishment stereotaxic coordinates followed those of our previous study [24].

### 4.3. Electroacupuncture (EA)

Mice and rats were individually acclimated in rectangular observation boxes for at least 2 h and then anesthetized with isoflurane. EA stimuli in mice was given on the day before MPTP injection (at 24 h intervals for a total of five days, Figure 1A). In rats, EA was given on the day after MPP^+^ injection (at 24 h intervals for a total of seven days, Figure 2A). Under anesthesia, a pair of stainless steel acupuncture needles (Tianjin HaingLimSou Won Medical CO., Ltd., Tianjin, China; Guage 40) was inserted 4–5 mm deep at the murine equivalent of the human GV34 and LR3. GB34 is located at the intersection of lines from the anterior borders to the head of the fibula; LR3 is located between the first and second metatarsal bones, 0.5 mm proximal to the margin of the web (Figure 1A) [29]. EA stimuli were delivered by an EA Trio 300 stimulator (Ito Co., Ito, Japan) at 1 mA intensities for 20 min duration at a frequency of 0 or 50 Hz, with a pulse width of 150 µs.

### 4.4. Rotarod Testing in Mice and Apomorphine-Induced Rotation Behavior and Locomotor Activity in Rats

The rotarod test was used to measure balance and motor coordination in mice. Before EA stimulation and MPTP injections, rotarod testing recorded the length of time a mouse stayed on a rotating rod (Ugo Basile S.R.L., Monvalle, Italy) with auto acceleration from 0 rotation per minute (rpm) to 40 rpm in 5 min (every 10 s plus 5 rpm), to obtain latency-to-fall baseline values. On the last day (day 8), rotarod testing was performed again to study the effect of EA stimulation. Eight days after MPP^+^ administration in rats, the effects of EA on motor asymmetry were examined via apomorphine-induced rotation behavior and locomotor activity (5 mg/kg, i.p.; Sigma-Aldrich). Rotation to the lesioned side within 30 min was measured according to the method described in our previous study [24]. The net rotation asymmetry score is expressed as the number of full body turns per minute. Apomorphine-induced locomotor activity was recorded for 30 min as the total movement distance (mm) by an automated system (Tru Scan system, Coulbourn Instruments, Whitehall, PA, USA).

### 4.5. Measurement of Dopamine Content by LC-MS/MS (Liquid Chromatography-Tandem Mass Spectrometry)

The striatum was dissected, weighed, and homogenized in 0.1 N perchloric acid, according to our previous study [24]. The supernatant was analyzed for dopamine content using LC-Q-TOF (MaxisImpact, Bruker, Billerica, MA, USA). Dopamine hydrochloride (pharmaceutical secondary standard; Sigma-Aldrich) was used for the analytical standard. Dopamine was quantified by the stable-isotope dilution method with the addition of d4-dopamine (Sigma-Aldrich).

### 4.6. Immunohistochemistry (IHC) and Western Blot

Brain tissue sections (30 μm in thickness) were pretreated with 3% hydrogen peroxide prior to incubation with 10% bovine serum albumin (BSA)/0.1% Triton X-100 in phosphate buffered saline (PBS) and anti-tyrosine hydroxylase antibody (MAB7566, Novus Biologicals, Littleton, CO, USA), and then with the biotinylated secondary antibody and avidin-biotin-peroxidase complex, as detailed in our previous study [24]. Labeling was revealed by treatment with 0.01% hydrogen peroxide and 0.05% 3,3′-diaminobenzidine (Sigma-Aldrich). Dissected rat substantia nigra was prepared as described previously [24]. Protein expression levels of pAkt, Akt, tyrosine hydroxylase, Bcl-2, pErk1/2, Erk1/2, BDNF, and β-actin in rat SN were determined by Western blot, according to our previous study [45]. Protein concentration was evaluated using the BCA protein assay kit (Pierce). Protein (20–40 µg) was separated using SDS-PAGE with a 10% or 12% resolving gel, and then electrotransferred onto Immun-Blot^®^ PVDF Membrane (Bio-Rad, Hercules, CA, USA). After blocking with 5% non-fat milk in tris-buffered saline (TBS) (20 mM Tris and 137 mM NaCl) for 1 h at room temperature, membranes were incubated overnight at 4 °C with pAkt (1:1,000, GTX128414; GeneTex, Irvine, CA, USA), Akt (1:1,000, #4691; Cell Signaling, Danvers, MA, USA), Bcl-2 (1:1,000, NB100-56098, Novus Biologicals), tyrosine hydroxylase (1:1000, MAB7566, Novus Biologicals), Erk1/2 (1:1000, GTX59618; GeneTex), pErk1/2 (1:1000, sc-7383; Santa Cruz Biotechnology, ; Santa Cruz, CA, USA), or BDNF (1:1000, ab108319; abcam) antibody diluted in TBS, then membranes were probed with GAPDH (1:5000, GTX100283; GeneTex) or β-actin (1:5000, GTX109639; GeneTex) antibody as the internal control. Blots were incubated for 1 h at room temperature with an horseradish peroxidase (HRP)-conjugated secondary antibody (1:10,000, #7074 or #7076; Cell Signaling), then protein bands were detected and estimated as described previously [45].

### 4.7. Statistics

Results are expressed as the mean ± standard error of the mean (SEM), and analyzed with the student *t*-test by GraphPad Prism 5 to determine the between-group statistical significance. The difference is considered significant when *p* < 0.05.

## 5. Conclusions

Our findings demonstrate that EA (50 Hz), given either as a pretreatment or after the initiation of disease symptoms, alleviates pathological changes in in vivo MPTP or MPP^+^ rodent models. These findings are encouraging for patients with PD.
